# Improved Patient Outcomes by Normalizing Sympathovagal Balance: Differentiating Syncope—Precise Subtype Differentiation Leads to Improved Outcomes

**DOI:** 10.1155/2018/9532141

**Published:** 2018-05-16

**Authors:** Nicholas L. DePace, Julie A. Bateman, Michael Yayac, John Oh, Mushfiqur Siddique, Cesar Acosta, Jeysel M. Pinales, Aaron I. Vinik, Heather L. Bloom

**Affiliations:** ^1^Department of Clinical Medicine, Hahnemann Hospital, Drexel University College of Medicine, 438 Ganttown Rd., Ste. B8-B9, Sewell, NJ 08080, USA; ^2^Department of Medicine, Pathology, and Neurobiology, Research and Neuroendocrine Unit, The Strelitz Diabetes Center, Eastern Virginia Medical School, 855 W. Brambleton Ave., Rm. 2018, Norfolk, VA 23510, USA; ^3^Department of Cardiac Electrophysiology and Medicine, Atlanta VAMC, Emory University School of Medicine, 1670 Clairmont Rd., Decatur, GA 30033, USA

## Abstract

Syncope is difficult to definitively diagnose, even with tilt-table testing and beat-to-beat blood pressure measurements, the gold-standard. Both are qualitative, subjective assessments. There are subtypes of syncope associated with autonomic conditions for which tilt-table testing is not useful. Heart rate variability analyses also include too much ambiguity. Three subtypes of syncope are differentiated: vasovagal syncope (VVS) due to parasympathetic excess (VVS-PE), VVS with abnormal heart rate response (VVS-HR), and VVS without PE (VVS-PN). P&S monitoring (ANSAR, Inc., Philadelphia, PA) differentiates subtypes in 2727 cardiology patients (50.5% female; average age: 57 years; age range: 12–100 years), serially tested over four years (3.3 tests per patient, average). P&S monitoring noninvasively, independently, and simultaneously measures parasympathetic and sympathetic (P&S) activity, including the normal P-decrease followed by an S-increase with head-up postural change (standing). Syncope, as an S-excess (SE) with stand, is differentiated from orthostatic dysfunction (e.g., POTS) as S-withdrawal with stand. Upon standing, VVS-PE is further differentiated as SE with PE, VVS-HR as SE with abnormal HR, and VVS-PN as SE with normal P- and HR-responses. Improved understanding of the underlying pathophysiology by more accurate subtyping leads to more precise therapy and improved outcomes.

## 1. Introduction

The current standard for diagnosing syncope is a positive tilt-table test performed according to one of the currently acceptable methods [1–5]. Presyncope and true syncope are difficult to definitively diagnose, even with tilt-table testing. Tilt-table testing may be useful for certain diagnoses, such as vasovagal syncope (VVS), neurocardiogenic (NCG) syncope, and postural orthostatic tachycardic syndrome (POTS). However, there are subtypes of VVS associated with autonomic conditions for which tilt-table testing is not useful in distinguishing. Tilt-tests, beat-to-beat (btb) blood pressure (BP) measurements, or in simpler form, pulse wave velocity measurements are also standards for autonomic testing. While btbBP is simpler to implement than tilt-table, it is often used in conjunction with tilt-table and, like tilt-table results, requires waveform assessment. Without overt symptoms, both are qualitative, subjective assessments, even in the hands of experts. Furthermore, measuring btb intervals from the BP waveform, with its much more rounded peaks as compared with the EKG waveform, introduces additional errors in the btb analyses. Heart rate variability (HRV) analyses (based on the EKG) also include too much ambiguity.

A more quantitative and universal method of assessing autonomic state is applied to both improve the diagnostic yield of syncope and provide a simpler (quantitative) diagnostic criteria, especially for the nonspecialist. This method improves the differentiation between the parasympathetic and sympathetic (P&S) activity. This method, when used in response to postural change or standing, helps to differentiate four etiologies underlying dizziness and lightheadedness: (1) parasympathetic excess (PE, associated with vagal symptoms), (2) sympathetic withdrawal (SW) associated with orthostatic dysfunction [6, 24], (3) sympathetic excess (SE) associated with (pre)syncope [7], and (4) vestibular dysfunction, a diagnosis by omission since it is not an autonomic dysfunction. Sympathetic excess (SE) associated with (pre)syncope is the topic of this report.

Defined herein are three of the subtypes of SE as manifested in VVS and NCG syncope. They are based on the differences in pathophysiology present in the P&S nervous system responses. Improved understanding of the underlying pathophysiology demonstrates how this more accurate subtyping leads to more precise medical therapy and thus improved patient outcomes. In general, an S-excess (SE) response to the stand challenge is associated with syncope. Abnormal sympathetic responses to stand differentiate syncope (SE) from orthostatic dysfunction (e.g., POTS; SW) [8, 24]. The subtypes of syncope are defined by the demonstration of P-excesses (PE) somewhere during the clinical challenge [9] or an abnormal HR response to stand. The three subtypes of VVS and NCG syncope are as follows:

1. SE + PE, which is VVS due to PE (VVS-PE), is defined as the presence of SE upon standing with PE demonstrated during rest, Valsalva, or stand, regardless of the HR response to stand.
2. SE + abn-HR, which is VVS with abnormal HR response (VVS-HR), is defined as the presence of SE upon standing with an abnormal HR response to stand (stand HR compared with resting HR).
3. SE (alone), which is VVS without PE (VVS-PN; “PN” for normal P-response), is defined as only the presence of SE upon standing. In these cases, the patients do not demonstrate an abnormal HR response to stand nor PE.

Using these subtype definitions, the P&S measurements from patients diagnosed with VVS-PE and VVS-HR are presented.

## 2. Methods

A database of 3670 consecutive, serial patients was followed in a large cardiology practice drawing from both urban and suburban populations. P&S function was assessed noninvasively using the ANSAR Medical Technologies, Inc. (Philadelphia, PA) software (ANX 3.0 autonomic function monitor). The ANX 3.0 computes simultaneous, independent measures of P&S activity based on continuous, time-frequency analysis of HRV with concurrent, continuous, time-frequency analysis of respiratory activity (RA). Time-frequency analyses employ a normalized CMORL wavelet with a Q of 5 and a spectral update of 4 seconds.

While this method facilitates reading P&S responses in the presence of arrhythmia [10], to permit comparison with standard HRV responses, 943 patients were omitted from this database due to high burden of ectopy (a run of more than two consecutive arrhythmic heart beats). Of the remaining patients, 2727 (50.5% female; average age: 57 years; age range: 12 to 100 years) were followed with more than one assessment over four years (an average of 3.3 assessments per patient). The mean time between assessments is 442.7 days. The patients carry diagnoses of cardiovascular disease (CVD) or a condition at high risk of future CVD, such as hypertension (32.7%), heart failure (35.2%), history of MI (16.2%), type 2 diabetes (36.2), renal disease (17%), or COPD (8.7%). The patients are on standard therapy [11].

**Figure 1:**
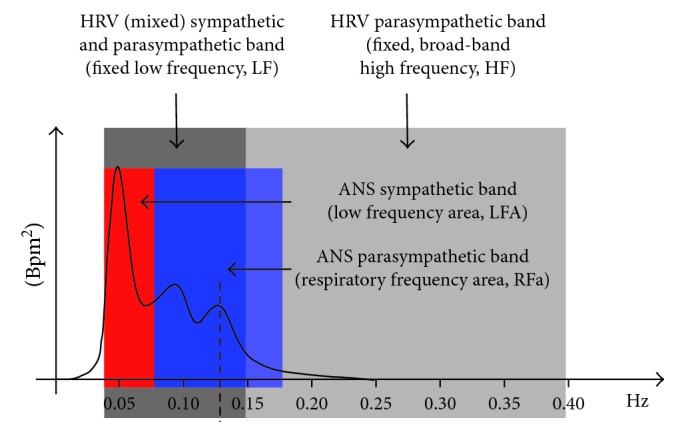
A spectral domain comparison of the LFa and RFa method [8] and the LF and HF method [12, 13] (see Methods for abbreviations). The vertical broken line represents the respiratory frequency over the four-second measurement period. The respiratory frequency is independently computed in the respiratory activity spectrum (not shown) and then transferred here to the HRV spectrum to locate the RFa (parasympathetic) spectrum. In this way, the RFa spectrum is based on the breathing rate of the subject. In this example, the respiratory frequency is 0.125 Hz (equivalent to 7.5 breathes per minute). The LF spectrum is represented in dark grey from 0.04 Hz to 0.15 Hz [12, 13]. The HF spectrum is represented in light grey from 0.15 Hz to 0.40 Hz [12, 13]. The RFa spectrum, in this example, is from 0.065 Hz to 0.185 Hz [8]. The RFa is computed from a frequency range centered on the respiratory frequency (0.125 Hz, see above) and moves as the respiratory frequency moves [8]. The LFa spectrum, in this example, is from 0.04 Hz to 0.065 Hz. The LFa is computed as the (fixed) LF frequency range (0.04 Hz to 0.15 Hz) minus the portion of the RFa frequency range that overlaps the LF frequency range (in this example, 0.065 Hz to 0.15 Hz) [8]. LFa, in (beats per minute)^2^ or bpm^2^, represents sympathetic activity, and RFa, in bpm^2^, represents parasympathetic activity [8, 14–17].

HRV-alone analyses compute mixed measures of P&S activity. For example, spectral HRV analyses result in a low frequency (LF) and a high frequency (HF) term [12, 13]. LF is a mix of both P&S activity (Figure 1) unless the subject's breathing rate is greater than about 13 breaths per minute [12, 13]. HF is a broad-band term [12, 13] (Figure 1), more than twice as broad as the known parasympathetic frequency range [14–19]. Therefore, even if the subject's breathing rate is >13 breathes/min, the HF term is mixed with noise, including harmonics. Both LF and HF require assumption and approximation to specify the P&S activity.

To eliminate the need for assumption and approximation required by LF and HF, independent spectral analyses of RA are added to spectral analyses of HRV [23]. This second independent P&S measure (RA) satisfies the algebraic requirement for a system with two independent components, fully characterizing the system, eliminating the need for assumption and approximation. Wavelet analysis eliminates the time-frequency approximations required by Fourier transforms and enables a significantly shorter data collection time to compute P&S activity. This enables autonomic transients and the dynamic activity of P&S interactions to be captured and analyzed. The resulting P&S terms are respiratory frequency area (RFa) and low frequency area (LFa), respectively, and sympathovagal balance (SB = LFa/RFa) is computed as a true ratio of independent parameters [8]. See the differences between LF and HF and LFa and RFa in Figure 1 [8].

The clinical study employed to determine P&S activity includes four well-known autonomic challenges, separated by resting baseline periods. These six periods are labeled in the figures as (A) resting baseline, (B) deep breathing, (C) baseline, (D) Valsalva maneuvers, (E) baseline, and (F) stand (postural change). The stand challenge, in the clinical study used in this article [9], is a postural change challenge, which is equivalent to tilt-testing [20]. The stand challenge is a physiologic activity and therefore inherently safer and more comfortable for the patient, arguably leading to more reliable results [7, 21]. The stand challenge enables autonomic testing to be performed in smaller clinics and in shorter time periods. From a safety point of view, the independent measures of P&S activity obviate the need for overt symptoms to be demonstrated, thereby inherently improving the safety of the study.

The time requirement, as well as the safety factor, is further improved with the implementation of a spectral analysis technique that eliminates the time-frequency compromise: the wavelet transform [18, 19, 22–29]. P&S monitoring [30] employs the wavelet transform, along with the appropriate time and safety considerations. Noninvasive BPs were taken during each phase of the clinical study [9]. This is an observational study. Patient testing and clinical outcomes measures were collected as an authorized part of the subjects' care and treatment given their clinical history. All data were handled in accordance with HIPPA regulations. Data were analyzed, statistically, with SPSS v 22.0, with the null hypothesis indicating significance at *p* ≤ 0.05.

## 3. Results

**Figure 2:**
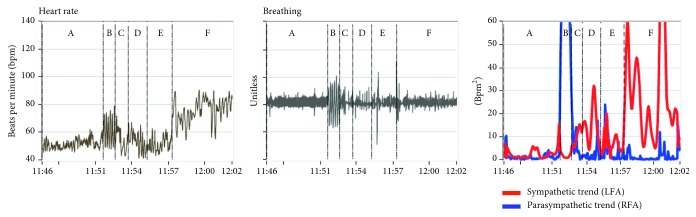
Instantaneous HR, instantaneous breathing, and instantaneous P&S responses (trends) to the standard clinical study from a syncopal, 23-year-old male. Note the instantaneous S-excess (SE, red trace) during the stand challenge (section “F”) of the trends plot (right plot). The SE correlates with the abnormal HR response to stand (left plot). HR remains high during stand, and there is no return to baseline. See text for details and Methods for abbreviations.

**Figure 3:**
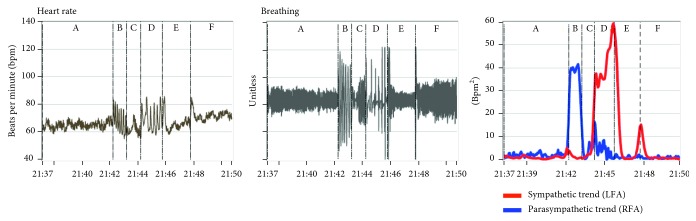
Instantaneous HR, breathing, and P&S responses (from left to right) to the standard clinical study from a healthy, 44-year-old female. Her average resting responses are a HR of 65 bpm, BP of 102/59 mmHg, S-activity (LFa) of 1.65 bpm^2^, P-activity (RFa) of 1.66 bpm^2^, and an SB (LFa/RFa) of 1.00 (unitless). See text for details and Methods for abbreviations.

Figure 2 presents a patient's responses to the standard clinical study, including instantaneous HR, breathing, and P&S data (parasympathetic trend (blue) and sympathetic trend (red) plot). The patient was previously diagnosed with syncope based on a positive tilt-test. For comparison, Figure 3 presents a normal subject's responses. Note that, while the resting (A) and paced, or deep, breathing (B) sections of the plots are similar, the Valsalva (D) and standing, or postural change (F), sections are not. In fact, they are essentially the opposite of each other. For the normal subject, the Valsalva S-response is significantly greater than that for stand. This is as it should be given that a series of short Valsalva maneuvers should induce a significantly greater physiologic stress response than changing posture from sitting to standing. However, for the syncope patient, the S-response to stand is greater than that for the Valsalva challenge.

The instantaneous SE, as seen in the trends plot of Figure 2, is important to note because there are cases, especially younger patients, where the average S-response to stand is normal. Meanwhile, these patients complain of lightheadedness (LH), and their instantaneous S-activity in response to stand is similar to that from the patient in Figure 2. This patient's peak S-response (the red curve) during stand (section “F” in the trends plot) is greater than the patient's peak S-response during Valsalva (section “D” in the trends plot). This is an “instantaneous SE” and is also associated with syncope, including tilt-positive patients [8]. The normal peak S-response relationship between Valsalva and stand is greater than 3 : 1, as exemplified in Figure 3.

These differences are also reflected in the heart rate response plots (Figures 2 and 3). The normal subject's instantaneous HR response to stand peaks during the gravitational reflex (during the first 30 seconds of stand) and quickly returns to resting baseline levels and remains near resting levels for the duration of quiet standing (Figure 3). However, the syncope patient's instantaneous HR response also peaks during the gravitational reflex but does not return to resting levels (Figure 2). Thereafter, the syncope patient's instantaneous HR tends to remain high (as compared with the resting response) and often begins to rise again and may continue to rise throughout quiet standing.

**Figure 4:**
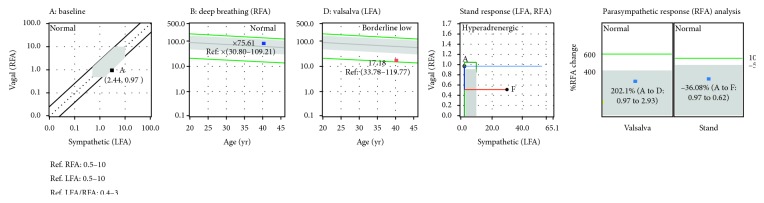
Average challenge responses from instantaneous responses in Figure 1. Patient's average resting responses included a HR of 84 bpm, BP of 109/73 mmHg, S-activity (LFa) of 2.38 bpm^2^, P-activity (RFa) of 0.68 bpm^2^, and a sympathovagal balance (LFa/RFa) of 2.49 (unitless). All average responses are within published normal limits (as represented by the grey areas) [5], except the stand response which indicates SE. See text for details and Methods for abbreviations.

Figure 4 presents the response plots from the syncope patient in Figure 2. These plots quantify the patient's average responses to challenge for comparison against published normal (average) ranges [9]. The response plots are to the clinical study's four challenges (as labeled: baseline (rest), deep breathing, Valsalva, and stand). Again for this syncope patient, the average responses over the 5-minute, resting, baseline, and 1-minute, deep breathing challenges are within normal limits (the grey areas on the plots). Note that deep breathing and Valsalva normal ranges are age as well as baseline adjusted. The patient's Valsalva response is low for the patient's age, as noted above. On average, over the 5-minute stand challenge, the normal P-response to stand is a decrease (any decrease) with respect to stand. The normal S-response to 5-minute stand is a 10% to 500% increase over rest [31] (Figure 3). An S-increase in response to stand greater than 500% is excessive (sympathetic excess, or SE) and is associated with syncope [9]. The stand response plot in Figure 4 demonstrates SE, reflecting the trends plot findings above (Figure 2).

Within this cohort at baseline, 38.6% of patients complain of lightheadedness (LH, not of vestibular etiology). Of the cohort, 31.4% (81.3% of the LH patients) were diagnosed with some form of orthostatic dysfunction, including POTS. Orthostatic dysfunction is associated with sympathetic insufficiency, or sympathetic withdrawal, upon standing (a decrease in S-activity from baseline (rest) to stand (postural change)). Of the remaining 7.2% of those complaining of LH, 3.9% were diagnosed with syncope (tilt-positive) or presyncope from other clinics. From the entire cohort, 5.2% of the patients demonstrate SE upon stand. All of these patients complained of LH, and all of the patients diagnosed with (pre)syncope elsewhere demonstrated stand SE.

**Figure 5:**
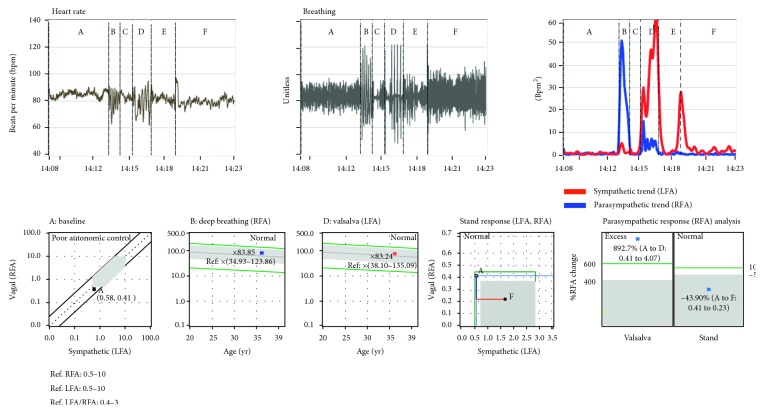
Results from a 34-year-old male, non diabetic patient, with a BMI of 25.7/in^2^, treated for labile hypertension with complaints of lightheadedness. At rest, his HR was 85 bpm, BP was 132/89, LFa was 0.58 bpm^2^, RFa was 0.41 bpm^2^, and SB was 1.41. At rest, he demonstrates advanced autonomic dysfunction (from the first plot on the second row, his response (point “A”) is below the grey, or normal, area due to his RFa being less than 0.5 bpm^2^) and PE with Valsalva (left panel of the last plot on the second row). From his trends plot (the last plot on the first row), his peak (red) S-response to stand (section “F”) is greater than one-third of that of Valsalva (section “D”), indicating an instantaneous SE, associated with (preclinial)syncope. Taken together, the SE with PE, VVS-PE is diagnosed. Treating the Vagal component and history of hypertension with Carvedilol [23] relieved both the syncopal events and the labile hypertension.

From Figure 2, instantaneous SE is demonstrated in the stand portion of the trends plot and supported by the SE demonstrated in the stand response plot, as in Figure 4. Again, this is not always the case as shown in Figure 5. In fact, many younger (12 to 30 years old), otherwise healthy patients complaining of LH present with similar results, and most of these are tilt-negative [8]. Younger (healthier), tilt-table-positive, syncope patients, often demonstrate normal, average autonomic responses (see the stand response plot in Figure 5); however, the instantaneous S-response to stand is comparable to (less than a 3 : 1 ratio) or greater than that to Valsalva (see the S-response curve in the Trends plot in Figure 5). Again, physiologically, the Valsalva challenge is a much more strenuous S-challenge than stand should be, at least three times as great [8]. This is a function of the fact that the average S-response to stand is averaged over 300 seconds, whereas the average S-response to Valsalva is averaged over 90 seconds. In many presyncope patients, the syncope indication is averaged out of the average stand response and must be visualized from the trends plots of instantaneous P&S responses (Figure 5).

**Figure 6:**
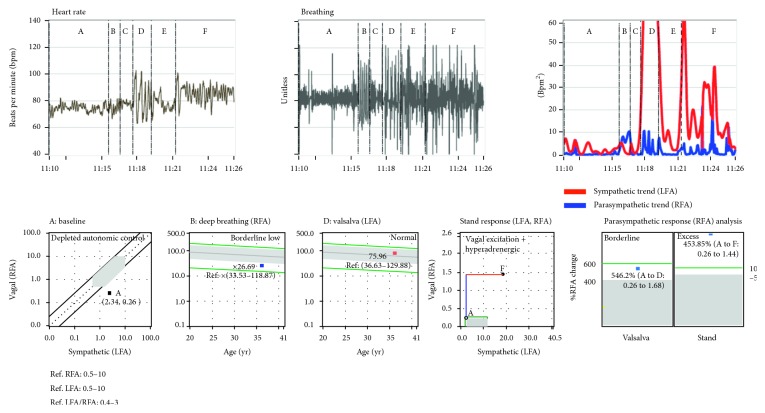
Clinical autonomic study results from a 36 year-old-male diagnosed with posttraumatic stress disorder and hypertension, with a BMI of 54.2/in^2^, and tilt-positive for vasovagal syncope. At rest, his HR was 75 bpm, BP was 147/94 mmHg, LFa was 2.34 bpm^2^, RFa = 0.26 bpm^2^, and SB was 9.16. At rest, he demonstrates advanced autonomic dysfunction (the first plot on the second row, his response (point “A”) is below the grey, or normal, area due to his RFa being less than 0.5 bpm^2^) and PE with Valsalva and stand (left panel of the last plot on the second row). From his trends plot (the last plot on the first row), his peak (red) S-response to stand (section “F”) is greater than one-third of that of Valsalva (section “D”), indicating an instantaneous SE, associated with (preclinical) syncope. Taken together, the SE with PE, VVS-PE is diagnosed. Treating the vagal component with the hypertension with carvedilol [23] prevented syncope and reduced his resting BP.

Figure 6 depicts a previously diagnosed, tilt-positive, VVS patient. In this case, not only is the average S-response excessive (see the red portion of the curve in the stand response plot), but so is the instantaneous S-response (see the red waveform in the Valsalva (“D”) and stand (“F”) portions of the trends plot). This is vasovagal syncope. The vagal component, in this case, is specified in the P-response to stand. The P-response to stand is indicated in two places. It is the blue portion of the curve in the stand response plot, and it is indicated in the right hand panel of the parasympathetic response analysis plot. In this case (Figure 6), a PE is indicated. Any increase in P-activity with standing is known to be abnormal [31]. The two indications combine to indicate VVS, specifically VVS-PE. Contrast Figure 6 with Figure 5. The latter demonstrates PE during Valsalva (see the parasympathetic response analysis plot in Figure 5). Both are examples of VVS-PE. Contrast these two figures with the first patient discussed and represented in both Figures 2 and 4. The first patient does not demonstrate PE; rather, an abnormal HR response is demonstrated. This with stand SE indicates VVS-HR.

## 4. Discussion

One of the most difficult forms of syncope to diagnose is VVS. Often tilt-table testing itself causes patients worry, anxiety, or stress. This stress (an S-stimulus) changes the patient's typical physiological response, the Vagal (or P-) excess, associated with VVS. In effect, the patient is temporarily treated by being placed on the tilt-table. As a result, the tilt-test may be falsely negative [8]. From the above, syncope with PE may be separately demonstrated, without overt symptoms. Then, if VVS-PE is demonstrated (preclinical), VVS subtype is confirmed. PE may be demonstrated during one or more of three challenges (Figures 5 and 6). PE may be demonstrated at rest as SB < 0.4. PE may be demonstrated with Valsalva as shown in Figure 5. PE may be demonstrated upon standing (postural change), as shown in Figure 6. All three, with stand SE, indicate VVS-PE.

SE upon standing is hypothesized as a result of the patient's brain becoming hypoperfused, which in turn causes an increase in S-activation in an attempt to normalize brain perfusion. The oscillations in the instantaneous S-activity demonstrated in the trends plots of Figures 2 and 6 may be the result of the patients' struggle to supply blood to the brain while being upright. The instantaneous SE, as seen in the trends plot, is important to note because there are cases, especially younger patients, where the average S-response to stand is normal. Meanwhile, these patients complain of lightheadedness (LH), and their instantaneous S-activity in response to stand is similar to that from the patient in Figure 2.

Given the difficulty of differentiating VVS from POTS, adding another parameter improves this differentiation. Again, stand SE is associated with syncope and SW is associated with orthostatic dysfunction [6]. In this way, VVS (indicated with SE) is reliably differentiated from POTS (indicated with SW). From the cohort, SE versus SW helps to improve differential diagnosis, including diagnosing presyncope where no cause of LH was determined. Corresponding modifications in therapy to properly address SE or SW, history dependent, helped to confirm the diagnoses, and in many cases, once the S-dysfunction was relieved, patients were weaned of their autonomic therapy. Often patients, especially older patients, have more than one pathology underlying LH, including both SW and instantaneous SE upon standing (DePace, personal communication). Identifying both enables simultaneous treatment of both.

BTB analyses, including heart rate variability (HRV), may be quantified with spectral analyses and other methods [12, 13]. However, care must be taken as to the selection of the protocol, the analysis technique, and the time duration over which data are collected. All choices impact the mathematical requirements, especially for the spectral analysis technique. The standard tilt-test, including with btbBP recordings, does not satisfy the mathematical requirements for standard spectral techniques (i.e., Fourier transforms or fast Fourier transforms (FFTs) [12]) and is a reason for the need to assess waveforms. A significant limitation of the Fourier transform is its inherent time-frequency compromise, forcing assumption and approximation to theorize activity specific to the parasympathetic and sympathetic branches of the autonomic nervous system, thereby reducing specificity and repeatability [32].

Differentiating the underlying abnormalities of the autonomic nervous system into specific subtypes based on pathophysiology significantly aids in therapy planning. In cases of VVS-PE, it has been found that PE should be treated as the primary autonomic disorder to effectively treat symptoms and underlying autonomic dysfunction. It is known that the parasympathetics set the threshold around which the sympathetics react. By treating the parasympathetics as the primary autonomic dysfunction and normalizing them, often the reactionary sympathetics (e.g., the SE) is naturally relieved, followed by BP or HR. When total relief is not experienced, what remains is a function of end-organ disorder (including vestibular) and typically requires less therapy.

In patients who are diagnosed with autonomic neuropathy or autonomic dysfunction and also heart diseases, hypertension, CAD, heart failure, or post-MI, the recommended therapy to treat both PE with SE and the cardiology diagnoses is carvedilol [33]. Carvedilol has a double effect, with both beta-blocker and alpha-blocker components and in low doses has antioxidant properties. In the presence of autonomic neuropathy, carvedilol seems to indirectly affect P-activity [34]. For VVS-PE without additional autonomic neuropathy, very low-dose anticholinergic therapy (e.g., tricyclics or SNRIs) is recommended to treat the PE [35].

In summary, traditional testing modalities (i.e., btb-cardiac activity measures in response to postural change, including tilt-table testing or standing) are confounded due to their measures mixing both P- and S-activity in a single parameter. Frequency analysis of standard btb-cardiac activity (with HRV or btbBP) in response to the stand challenge is further compromised by the nature and definition of Fourier transforms or fast Fourier transforms (FFT), including short-term FFT (st-FFT) [12]. Fourier transforms, of any sort, carry the mathematical requirement of long-duration, stationary (or stable) signals. Signal stationarity requires that the characteristics of the signal not change significantly (remain quiescent) over the analysis period.

During the course of the first five minutes after a head-up postural change (including sitting to standing), there are several physiologic changes that affect the stand response, including (1) the response to the gravitational challenge, (2) the response to the exercise reflex, and (3) the recovery from both. Even a normal gravitational response comes and goes over a 30 second period, invalidating the use of Fourier transforms. An abnormal response to any one of these three physiologic changes may underlie LH and lead to syncope. As a result of these changes, the stand challenge is not stationary (quiescent), even st-FFTs with a 32-second analysis window, as per the standard practice [12, 13]. Therefore, the use of the Fourier transform is inappropriate and nondiagnostic. Wavelet analyses address and avoid these issues by addressing time and frequency together, rather than attempting a compromise between time and frequency. Wavelets with independent RA analyses allow for independent computation of P- and S-activity, which in turn clarifies the actual underlying pathophysiology associated with VVS and its different subtypes, as described herein. The wavelet is valid in all instances, including those encountered during the clinical, autonomic assessment protocol employed in this study, which includes the stand or postural change challenge and its reflexes [25–29].

## 5. Conclusions

VVS-PE is perhaps the most common subtype of syncope. Further differentiating syncope by identifying the autonomic components helps to improve differential diagnosis, which improves therapy planning, resulting in improved outcomes. Independent P&S monitoring provides more specific data regarding the pathophysiology of VVS. Improved subtype differentiation allows for more precise therapeutic modalities and improved symptom management. In the case of VVS-HR, ruling out VVS-PN may lead to lower doses of medication prescribed while still improving patient outcome.
